# Evaluation of outpatients with only conjunctivitis for SARS-CoV-2 infection during COVID-19 pandemic

**DOI:** 10.5935/0004-2749.20210060

**Published:** 2025-02-02

**Authors:** Fadime Kendir Uguz, Aykut Demirkol, Yasemin Demirkol, Levent Akçay

**Affiliations:** 1 Department Of Ophthalmology, Atatürk Devlet Hastanesi, Antalya,Turkey; 2 Department Of Ophthalmology, Dünya Eye Hospital, İstanbul,Turkey; 3 Genomic Laboratory, Umraniye Teaching and Research Hospital, University of Health Sciences, Istanbul, Turkey; 4 Department of Paediatric Genetics, Umraniye Teaching and Research Hospital, University of Health Sciences, Istanbul, Turkey

## INTRODUCTION

The coronavirus disease-2019 (COVID-19), which originated in Wuhan in the Hubei Province of China, has spread rapidly all over the world^(1)^. It has been reported that about 0.8% of the COVID-19 patients experience conjunctival congestion^(2)^. This study aimed to check outpatients with only conjunctivitis during the pandemic for severe acute respiratory syndrome coronavirus 2 (SARS CoV-2) based on nasopharyngeal and conjunctival real-time reverse transcription-polymerase chain reaction (RT-PCR) positivity.

## METHODS

A prospective interventional case series study was designed. Outpatients who were confirmed to have conjunctivitis alone were included at the Dünya Göz Hospital as outpatients from May 15, 2020 to June 15, 2020. Forty-five patients with conjunctivitis and 33 age-and sex-matched controls were included in the study. Twenty-three (51.1%) patients had bilateral conjunctivitis. There were no statistically significant differences in the mean outpatient age and female/male ratio between the conjunctivitis and control groups (46.4 ± 14.9 years, range: 20-81; 47.5 ± 15.8 years, range 21-83; 24/21, 15/18, respectively, p=0.75). RT-PCR tests (Coyote Bioscience Co. Ltd) were performed with nasopharyngeal and conjunctival swab samples. All patients were treated with topical lomefloxacin 4 times a day for 7 days, and the symptoms regressed.

The study protocol followed the tenets of the Declaration of Helsinki and was approved by the Ethics Committee of the Üsküdar University (61351342/2020-330). Written informed consent was obtained from all the patients in advance. All statistical tests were performed using the Jamovi project (2019) (Jamovi version 1.1, computer software). The data were presented as mean ± standard deviation.

## RESULTS

Table 1 shows the demographic characteristics of the outpatients and the controls. None of the conjunctival and nasopharyngeal swab samples were positive either in the outpatient group or in the control group. All outpatients and controls were questioned once a week for a period of two weeks regarding systemic symptoms of COVID-19. None of the patients and controls exhibited any symptoms of the disease such as fever, dry cough, dyspnea, fatigue, pharyngalgia, headaches, diarrhea, or myalgia.

**Table 1:** Demographic characteristics of the outpatients and control subjects

	Outpatients with negative RT-PCR results (n=45) and controls (n=33)
Age (years, mean ±SD)	46 ± 14.9; 47.5 ± 15.8
Sex M/F	21/24; 18/15
%	46.7/53.3%; 54.5/45.5%

## DISCUSSION

COVID-19 is mainly transmitted through infectious droplets from the patients and by direct contact with virus-contaminated fomites. If the ocular surface is directly exposed to infectious droplets and contaminated hands, the eye may serve as a point of entry for SARS-CoV-2^(3)^. In a case series, one-third of the patients with severe COVID-19 were found to have ocular manifestations^(4)^. In a meta-analysis involving 1167 Chinese COVID-19 patients, it was shown that conjunctivitis could be associated with a severe form of COVID-19^(5)^. In our study, all outpatients with only conjunctivitis as well the controls had no conjunctival or nasopharyngeal positivity for SARS-CoV-2 on RT-PCR. The small number of participants was a key limitation of this study. Based on the findings, it could be stated that during the COVID-19 pandemic, conjunctivitis alone is not a critical symptom warranting the evaluation of outpatients for SARS-CoV-2 via RT-PCR. However, further studies are required. To the best our knowledge, this is the first report on the evaluation of outpatients with only conjunctivitis for SARS-CoV-2.
